# Supplementary material for: Are carbon emissions trading and green financial instruments synergistic? -Comprehensive quantitative research based on content analysis
Source: PLoS One. 2024 Mar 7;19(3):e0298601. doi: 10.1371/journal.pone.0298601 (PMC10919601; doi:10.1371/journal.pone.0298601)
Supplement: S1 Data — (ZIP) [file pone.0298601.s001.zip › Data/地级市Z_F工作报告.zip!/─■╧─/╥°┤¿╩╨/└┤╫╘╣1⁄2╓┌║┼í╛┬φ┐╦╩2╛▌═°í┐_2018.docx]

各位代表：

现在，我代表市人民政府向大会作政府工作报告，请予审查，并请市政协委员和其他列席会议的同志提出意见。

一、2017年工作回顾

过去一年，面对复杂多变的外部环境和持续加大的经济下行压力，在自治区党委、政府和市委的坚强领导下，在市人大及其常委会和市政协的监督支持下，市政府团结带领全市人民，把握新常态，贯彻新理念，积极应对各种困难和挑战，全力以赴推进发展。预计，全年地区生产总值增长8.0%；规模以上工业增加值增长8.5%；全社会固定资产投资增长1.0%；社会消费品零售总额增长9.5%；地方财政一般公共预算收入同口径增长9.2%；城乡居民人均可支配收入分别增长8.1%和8.5%；外贸进出口总额增长55%。三度蝉联“全国文明城市”，第八次获评“全国双拥模范城市”，入选首批十大“中国旅游休闲示范城市”，喜获“全国幸福城市”“中国领军智慧城市”等10多项国家级殊荣，银川的知名度、美誉度大幅提升。

一年来的主要工作是：

——经济发展质量进一步提升。坚持创新引领，加快转型升级，出台“创新驱动三年行动计划”，精准打好“工业19条”“房地产16条”“科技强市17条”等政策组合拳。组建银川产业技术研究院，搭建创新平台111个，引进培养急需紧缺人才2000多名。新增一批“专精特新”中小企业，引进比亚迪新能源汽车项目，高端装备制造业向智能化、绿色化迈进，国家智慧型纺织基地初现规模，百亿级晶体材料产业集聚发展，高技术产业增加值增长30%。农业优势特色产业产值比重达到90%以上，优质蔬菜、乳品和葡萄酒、花卉等特色农产品出口30多个国家和地区，农业嘉年华等活动引领创意休闲产业蓬勃发展，跻身国家休闲农业乡村旅游示范市。国家现代服务业综合试点成效明显，“欢乐购物季”等活动带动消费升级，“消费在银川”品牌效应进一步凸显，会展经济持续升温，网络零售交易额增长20%，社会物流总额增长17.6%，文化消费增长15%。全域旅游核心区建设扎实推进，游客接待量、旅游收入分别增长29.7%和22.9%。41家知名互联网企业集聚银川，数字经济、共享经济不断壮大。

——改革开放活力进一步迸发。坚持以改革促开放，以开放促发展，56项改革任务全面落实。供给侧结构性改革取得实效，淘汰落后产能10万吨，万元GDP能耗下降5%，待售商品房去化周期缩短至12个月，降低企业电力、税费、制度性交易等成本9亿多元。“放管服”改革持续深化，实现证照电子化和网上审批100%，“不见面”审批事项达到80%，审批时限提速88%，审管互动、监管联动日趋成熟，新增企业、注册资本分别增长13.41%和3.89%。金融改革效果显著，25支产业基金撬动346亿元社会资本投入实体经济，西部担保公司担保额突破240亿元，通联资本公司成功发行全区首单境外债券3亿美元,创设西北首家股权科技板，金融业增加值占三产比重达到26%。农业农村改革步伐加快，农村土地承包确权通过自治区验收，土地股份经营、陆基生态渔场等新模式助推农村经济发展。公立医院改革得到国务院肯定。国资国企、文化教育、社会保障等领域改革稳步推进。“两园三区”开放平台加快建设。中关村双创园引进创新型企业24家，银川·中关村创新中心挂牌运营；丝路经济园引进总部经济、金融商贸等高轻新项目20个；经开区与北京经开区共建“一带一路”国际产业园，引进石墨烯等高端项目6个；滨河新区现代纺织、大数据、生命健康等产业集聚发展；综保区国际快件、裘皮集散、棉花外发加工等功能不断拓展，通关时间压缩70%。开放通道更加畅通。新增国内国际航线13条，旅客吞吐量突破800万人次。月牙湖通航机场开航运营。开通银川至德黑兰国际货运班列，出口货值4200万美元。对外经贸合作取得新进展。引进外资20.51亿美元。设立驻沙特（吉达）经贸联络处，中沙产能合作进入实质性阶段。加强与京津冀、长三角地区及全国工商联会员单位合作交流，招商引资到位资金突破600亿元。

——城乡人居环境进一步改善。坚持城乡统筹，加快生态文明建设，出台“生态立市三年行动计划”，编制新型城镇化、海绵城市等各类规划35项，空间规划、“城市双修”、城市设计等国家级改革试点顺利推进。闽宁镇、掌政镇被评为全国第二批特色小城镇。高铁、公路、综合交通枢纽、地下综合管廊、“东热西送”等重点项目加快实施，新建改建城市道路（街巷）38条，改造棚户区7021套、老旧小区352万平方米、城市积水点19处，北京路、贺兰山路沿线违规“住改商”整改率达到75%。垃圾资源化利用率达到70%。智慧交通、智慧政务等智能化服务更加便民，银川入围国际标准化组织ISO智慧城市标准试点。城乡环境综合治理“九大行动”扎实推进，中央环保督察反馈问题抓紧整改，贺兰山东麓国家级自然保护区年度整治任务全面完成，转入生态修复、完善管理阶段。出台大气治理10条，建成全区首个大气监测超级站，改造淘汰燃煤锅炉599台，清查整治散乱污企业461家，更新新能源公交车500辆，投放共享单车3万辆，淘汰黄标车、老旧车辆1.5万辆，空气质量下滑势头得到有效遏制。强力开展冬季大气污染防治攻坚战，冬季优良天数达标率首次突破75%。788名河长领责护河，第七、九污水处理厂建成，第二、四、五污水处理厂完成提标改造，完成9条城市黑臭水体年度治理任务，整治湖泊湿地6800亩，全国水生态文明城市试点通过国家验收，提名国际湿地城市认证。实施生态绿化项目52个，造林5万亩，花博园和10个小微公园建成投用，人均公园面积、湿地面积分别达到16.52平方米和240平方米，居西部省会城市首位。成功举办第九届中国花卉博览会、TMF全球智慧城市峰会、亚洲都市景观奖颁奖礼等重大节会，向国内外全方位展示了银川形象。

——人民群众幸福感进一步增强。财政支出80%以上用于民生，20件民生实事全面完成。出台“脱贫富民三年行动计划”，扶贫保全覆盖，完成润丰村等4个安置点1556户7013人搬迁，1个贫困村和8216名建档立卡贫困群众脱贫销号，移民地区人均收入增长11.67%，分别高于城乡居民人均可支配收入3.57和3.17个百分点。就业创业扶持力度持续加大，建成创业孵化基地5个，创业带动就业19651人，新增城镇就业6万人，农村劳动力转移就业10.8万人，城镇登记失业率3.56%。城乡居民基础养老金最低标准提高至220元，高于西北其他省会城市。5.63万名被征地农民养老保险转入城镇职工养老保险。城乡居民医保财政补助提高6.4个百分点。在西部地区率先将因病、残疾照顾返贫致贫家庭纳入低保，城乡低保补助标准分别提高30%和20%。建成保障房6000套，发放廉租补贴1927万元，3万余名困难群众受益。公积金互助功能不断拓展，1.1万户家庭圆了住房梦。创新“首都带首府”教育、医疗合作模式。新建中小学、幼儿园16所，增加学位14500个，完成100所中小学信息化改造，购买151所普惠性民办幼儿园服务。出台健康银川实施意见，组建医疗联合体6个，引进互联网医疗企业20家，服务全国患者436万人次。成功举办首届银川国际马拉松、全国青少年阳光体育大会等全民健身活动，全民运动健康意识进一步增强。建成并免费开放基层综合性文化服务中心30个，开展文化惠民活动2700余场次。首届文化创意节、互联网电影节圆满落幕，赴国外文化交流50余场次，贺兰山文化等特色文化品牌影响力不断提升。“平安银川”建设深入推进，八类主要刑事案件立案数下降16%。安全生产四项约束性指标全面下降。食品药品安全保障水平持续改善，防灾减灾救灾和突发事件应急处置能力不断增强。城市公共安全指数位居全国35个主要城市第五。民族团结进步示范市创建工作扎实推进,宗教工作法治化水平显著提升。工会、青少年、妇女儿童、残疾人、红十字、档案、文史、普法、气象等工作取得新成绩。

——政府治理能力进一步提升。认真学习贯彻党的十九大、自治区第十二次党代会、十二届三次全会精神和市委十四届四次、五次全会精神，“两学一做”学习教育常态化制度化扎实推进，“四个意识”进一步增强，抓落实的能力得到提升。自觉接受人大法律监督，主动接受政协民主监督和社会公众监督，人大议案建议和政协提案办复率达100%。依法制定行政规范性文件13件、废止16件，主动公开政府信息10.8万余条。城市执法体制改革全面实施，80%执法力量下沉基层一线。严格落实中央八项规定精神、国务院“约法三章”和区市若干规定，全面加强行政监察、审计监督、执纪问责，查处违反中央八项规定精神案件43起问责29人，开展“电视问政”5期问责干部142人，解决群众关注的热点难点问题465件，问责“三不为”干部161人，政府工作效率和服务质量显著提升。被列为国务院第四次大督查实地免督查单位，荣获“中国政务服务突出贡献奖”。

各位代表，过去的一年，我们遇到的困难比预料的多，面对的挑战比预估的大，取得这样的成绩实属不易。这是自治区党委、政府和市委坚强领导的结果，是市人大、政协关心支持的结果，是全市上下务实苦干、攻坚克难、团结拼搏的结果。在此，我代表市人民政府，向全市人民，向给予政府工作大力支持的人大代表、政协委员，向各民主党派、工商联、各人民团体和社会各界人士，向驻银中央和自治区各单位，向驻银解放军、武警消防官兵、公安干警，向关心和支持银川改革发展的社会各界朋友们，表示崇高的敬意和衷心的感谢！

在看到成绩的同时，我们也清醒地认识到，对照中央“五位一体”总体布局和“四个全面”战略布局，对照自治区党委、政府“走在前、作表率”的要求，对照人民群众日益增长的美好生活需要，我们的工作还有不少差距，发展中的一些矛盾问题集中显现，主要是：经济总量不大，发展层次不高，面临着加快发展与提高质量、高端突破与补齐短板等诸多挑战；产业结构单一，发展倚重倚能，面临着污染落后产能退出与新旧动能转换不畅、产业转型升级与集群培育等诸多难题；城市发展模式粗放，功能配套还不能满足发展要求，面临着土地等存量资源利用效率不高与发展空间受限、城市承载力较低与城市运营成本较高等诸多矛盾；乡村建设滞后，改革发展任务繁重，面临着城乡发展不平衡与缩小城乡差距、乡村振兴与城乡一体发展等诸多压力；居民增收难度加大，民生保障水平不高，面临着投资不足与消费乏力、财力紧张与提高社会保障能力等诸多困难；发展对创新要素吸附力不强，高层次人才等资源严重短缺，面临着推动科学发展与干部能力不足、事业留人与招才引智等诸多考验。这些矛盾问题交织迭加，困扰和制约着银川的发展。特别需要说明的是，虽然经过不懈努力，去年固定资产投资、优良天数两项指标还是没有达到年初预期目标。在此，诚恳接受各位代表和全市人民的批评。我们将本着对党、对人民、对银川发展高度负责的态度，下大力气解决存在的问题，全力做好今后各项工作。

二、2018年工作安排

今年是贯彻落实党的十九大和自治区第十二次党代会精神的开局之年，是决胜全面建成小康社会、实施“十三五”规划承上启下的关键一年，同时迎来了改革开放40周年和自治区成立60周年。立足当前，展望未来，党的十九大开启了新时代中国特色社会主义新征程，自治区第十二次党代会描绘了未来五年全区发展的宏伟蓝图，特别是市委十四届四次、五次全会把握新形势、贯彻新部署、顺应新期待、追求新作为，提出了“绿色、高端、和谐、宜居”城市发展理念和在全区率先全面建成小康社会、率先开启全面建设社会主义现代化新征程奋斗目标，为银川加快发展指明了方向,是做好当前和今后一个时期工作的行动纲领。站在新时代的新起点，重新审视首府大环境、新优势，银川将迎来新一轮发展机遇期，只要我们奋发有为、敢为人先，就一定能够爬坡过坎、转型升级。奋进正当其时、发展正当其势。我们要牢固树立政治意识、大局意识、核心意识、看齐意识，紧紧团结和依靠全市人民，坚决克服惯性思维和路径依赖，拿出“人一之我十之、人十之我百之”的干劲，追求卓越、示范引领，切实把理念变成思路，把思路转化成行动，把行动落实成项目，把项目建设成工程，努力走出一条符合银川实际的高质量发展之路。

按照市委十四届五次全会部署，今年政府工作的总体要求是：高举中国特色社会主义伟大旗帜，以习近平新时代中国特色社会主义思想为指导，深入贯彻落实党的十九大、自治区第十二次党代会、十二届三次全会和市委十四届四次、五次全会精神，全面落实中央、自治区经济工作会议各项决策部署，坚持稳中求进、好中求快工作总基调，按照高质量发展和“走在前、作表率”的要求，坚持“绿色、高端、和谐、宜居”城市发展理念，以首府责任、首府标准、首府担当，深入实施创新驱动、生态立市、脱贫富民、乡村振兴发展战略，着力打好“三大战役”、实施“五大工程”、开展“六项计划”、推进“四项行动”，以新时代的新气象新作为，喜迎自治区成立60周年，奋力开创“两个率先”新局面。

全市经济社会发展主要预期目标是：地区生产总值、规模以上工业增加值、全社会固定资产投资、社会消费品零售总额均增长8.0%左右，地方财政一般公共预算收入增长7.0%左右，城乡居民人均可支配收入分别增长7.5%和8.0%，节能减排和环境质量完成自治区下达目标任务，努力在实际工作中争取更好结果。

（一）聚焦绿色发展，打好“三大战役”，加快建设西北地区生态文明先行市。着眼生态脆弱现状，深入实施生态立市战略，坚持“破”与“立”并重，打破资源环境约束趋紧瓶颈，加快形成绿色发展新格局，让绿水青山变成金山银山。

打好绿色生产战役。坚持绿色低碳循环发展，提高经济绿色化程度，向绿色要产品质量、要生活品质、要发展效益，决不要带污染的GDP。加快绿色生产体系建设。实施绿色制造三年行动计划，开展绿色园区示范创建，深入推进园区低成本改造，推行园区综合能源资源一体化解决方案，建设绿色管理服务平台，争创1家自治区及以上绿色园区。开展工厂用地集约化、生产清洁化、废物资源化、能源低碳化等绿色改造，争创5家自治区及以上绿色工厂。开展绿色供应链管理试点，加快建立以资源节约、环境友好为导向的采购、生产、营销、回收及物流体系。支持企业参与创建国家标准和行业标准，打造一批高质量的绿色产品。着眼于产业发展前沿，深入研究、大力培育发展新产业新业态新技术新模式，加快构建新兴绿色产业体系。推广绿色建筑和建材，发展装配式建筑。淘汰无效低效落后产能。制定产业引进和限制目录，严格执行环境保护、产品质量、安全生产等方面的法律法规和标准，淘汰活性炭、水泥等落后产能2万吨。坚持“一企一策”，逐步出清“僵尸企业”。严格能源消耗总量和强度双控制度，加快变频节能、余热回收等节能技术应用，完成污水处理设施技改、生物发酵企业尾气治理等20个以上清洁化改造项目。积极倡导绿色生活方式。广泛开展创建节约型机关、绿色家庭、绿色学校、绿色社区和绿色出行等行动，推进全民节能、节水，推广低碳交通，普及绿色消费，让勤俭节约、绿色低碳、文明健康的生活方式成为全市人民的行动自觉。

打好生态系统保护战役。山水林田湖草构成的塞上江南景观是大自然对银川的馈赠，凝结着历代银川人的辛勤劳动和付出。我们要将山水林田湖草作为一个生命共同体，加快生态保护和修复，林果结合、草畜结合、种养结合，推进生态治理、造林绿化、景观打造和农林牧渔业一体化发展，实现生态效益、社会效益、经济效益相统一。着力拓展绿色空间。严守生态保护红线、永久基本农田、城镇开发边界三条控制线，统筹推进经济林建设、绿化美化、防沙治沙、湿地保护、水土保持等工作，抓好贺兰山东麓国家级自然保护区银川段生态修复和长效管护，实施黄河两岸、艾依河两侧、贺兰山东麓生态林建设，提升生态系统质量和稳定性，切实维护好西北生态安全屏障。加强国土空间绿化。突出生态功能，兼顾景观功能、游憩功能、户外功能和经济功能，启动大规模国土绿化行动，引导社会各界参与生态建设，实施城市主干道景观提升等一批生态绿化项目，在城乡社区小区、企业机关、公园广场等空地，见缝插绿、植绿添绿，植树造林7.74万亩，建设10个小微公园，改造提升城市公园，争创国家森林城市，争取通过国际湿地城市认证。

打好环境问题整治战役。紧盯全面建成小康社会生态环境短板，全力加强污染防治，突出抓好大气、水、土壤等污染治理，全面完成中央环保督察反馈问题整改，着力控制污染源头，狠抓末端排放治理，让群众感受到环境改善的成果。抓好大气污染防治。坚决打赢蓝天保卫战，完成“东热西送”一期工程，全部拆除20蒸吨以下燃煤锅炉，力争建成区集中供热率达到75%。全面完成火电及企业自备燃煤发电机组超低排放改造，巩固药企异味治理成果，大力整治秸秆焚烧行为。严格落实建筑工地“6个100%”扬尘防控措施。深化“以克论净、深度保洁”模式，实施道路积尘考核，积极推进市区主干道路机械化清扫全覆盖，确保扬尘污染得到有效控制。推进老旧车辆淘汰，加强高排放车辆监管。新辟、优化调整、加密公交线路30条，更新一批新能源公交车，建设充电桩1185个，推广使用新能源汽车。力争空气质量优良天数比例达到75%，带给群众更多蓝天幸福感。抓好水污染防治。开展水环境治理三年行动和清河专项行动。深化保护母亲河、艾依河行动，全面消除城市黑臭水体，建成6条排水沟入黄截污净化人工湿地，黄河和艾依河银川段水质达到Ⅲ类。实施西北部区域防洪排涝及湖库水系综合整治工程，构建“六纵六横”河湖库连通水系。建成区污水全部接入处理管网，处理率达到98%，所有污水处理厂达到一级A排放标准。实施城市水源地替代工程，关停城市68眼自备井，搬迁、关停绕城高速以内“小散乱污”企业、污染源和禁养区内所有畜禽养殖场（养殖小区），让人民群众喝上干净水。抓好土壤污染防治。开展土壤污染状况详查，完成1033个土壤详查点位监测。建立土壤环境保护与污染治理项目库，实施土壤污染治理与修复技术应用试点项目。推进农业绿色发展“五大行动”“三减”计划，力争化肥农药减少20%，让人民群众“米袋子”“菜篮子”更安全。像对待生命一样对待生态环境，实行最严格的生态环境保护制度。健全环保信用评价、信息强制性披露、严惩重罚等制度，建立司法联动机制，以“零容忍”的态度惩处破坏生态环境行为，让银川天更蓝、地更绿、水更美、空气更清新。

各位代表，绿色是生命之源、发展之本。人与自然是生命共同体，人类对大自然的伤害最终会伤及人类自身。我们要始终保持绿色发展定力，把绿色理念融入决策、实践、发展的各个阶段，体现在生产、生活、生态的各个方面，共建共享山清水秀的优美环境，筑牢可持续发展的基础，切实让“风景”变成“产业”，将“美丽”变成“生产力”。

（二）聚焦高端发展，实施“五大工程”，加快建设国际化、现代化、智慧化城市。端正首府站位，激活后发优势，把握新时代经济发展特征，深入实施创新驱动战略，瞄准产业高端和高端产业，率先推动经济质量变革、效率变革、动力变革。

实施产业转型升级工程。坚持产业集群化、高端化、智能化发展方向，加快“互联网+”、大数据、人工智能与实体经济深度融合，推动主导产业全产业链发展和衍生产业、配套产业、关联产业统筹发展，着力提高产业规模层次和产品质量效益，抢占发展制高点，推动形成“10+X”产业发展格局，切实把每一项产业做成能够支撑发展、聚才引智、值得为之奉献的事业。高端装备制造业，瞄准中国制造2025，加快信息化、数字化、智能化改造步伐，大力推进智能铸造、智能仪表等产业项目，建设智能工厂、数字化车间5家，培育“两化”融合标杆示范企业10家。积极争取航空航天、国防科工等军工集团来银发展。现代纺织产业，依托国家智慧型纺织基地，加快构建从纺纱、面料、服装到终端市场全产业链，推动恒天如意12万吨氨纶、1亿米色织布等项目建成投产，培育2-3个高端品牌，推动科技时尚绿色纺织集群发展。新材料产业，推动光伏、晶体材料从多晶硅、硅片、拉棒、切片、电池及组件全产业链、集群化发展，加快石墨烯储能材料生产项目建设，抓好天通蓝宝石二期、银和大尺寸半导体、隆基硅二期等项目建设，打造晶体材料产业集群。新能源产业，实施宝丰光伏二期、德润葡光互补等项目，提升国家新能源综合示范城市品牌效应。现代服务业,着力向专业化、精细化和高品质转变，建设一批智慧商圈和特色街区，发展线上、线下相结合的“新零售”，办好“欢乐购物季”、汽博会、房博会等品牌活动和展会。大力发展研发设计、金融贸易、商务咨询、信息技术、知识产权服务等新业态。现代物流产业，以推进物流网络化、智能化、柔性化发展为重点，实施“互联网+”流通行动计划，完善冷链物流体系，加快智慧物流、标准化配送和追溯体系建设，打造西北地区物流枢纽。文化旅游产业，制定全域旅游实施意见。加快国家文化消费试点城市建设，创建特色文化产业示范基地，支持影视动漫、数字文化创意等新兴文化产业发展。推进丝路明珠塔、长城神秘西夏文创园等项目，发展大阅海旅游圈。推动西夏陵申遗、贺兰山岩画申报国家5A级景区。完善旅游基础配套，实施“旅游+”计划，开发旅游新产品新业态，推动旅游与文化、运动休闲、健康医养等业态融合发展。布局“银川故事”“新丝路驿站”推介银川，打响“丝路明珠·魅力银川”城市品牌。力争游客接待量和旅游收入增长15%以上。新能源汽车产业，以发展整车和关键零部件为重点，实施比亚迪纯电动乘用车、云轨、纯电动客车生产基地项目建设。生命健康产业，突出医养结合、康体保健，抓好滨河生命谷、宝丰健康城等项目建设，支持互联网医疗加快发展，高起点打造养老、医疗、康复为一体的健康产业基地。规划建设生物医药基地，支持开发生产特色高端原料药和仿制药。葡萄酒产业，充分发挥银川产业技术研究院科技引领推广作用，建立一体化全产业链技术标准体系和管理规范，培育龙头企业和知名品牌，发展壮大集种植、酿造、研发、品牌建设为一体的产业集群，办好贺兰山葡萄酒国际旅游节，打造国际葡萄酒之都。抓好传统产业改造提升，实施40个重大技改项目，推动能源化工、生物医药、羊绒加工等传统产业老树发新芽。大力发展大数据、云计算、电子商务、电子竞技、通用航空等新产业新业态，培育新的经济增长点。

实施园区转型发展工程。针对现有园区有数量缺质量、多而不强的现状，要按照“整合园区、盘活存量、低端退出、转型升级”的思路，推动园区高端高效高速发展。优化园区发展布局。以“两园三区”为重点，构建“一县一园、一园多区、各有特色”的发展格局，做强主业，提质增效。中关村双创园，重点链接北京中关村高端制造、研发孵化、科技金融等新兴产业，抓好宇联智能科技创新中心、集成电路产业研发中心、物美数据中心等项目，打造西部“中关村”。丝路经济园，重点发展以民营总部经济为引领的金融贸易、信息技术等现代服务业，引进民营企业总部。经济技术开发区，与北京经开区共建“一带一路”国际产业园，重点推动高端装备制造、新材料、生产性服务业等产业集群发展。滨河新区，与苏州工业园区合作共建飞地工业园，实施山屿海医养、钛酸锂电池等项目，全力推动“一核三业五谷”发展。综合保税区，整合临港经济区，重点发展现代纺织、黄金珠宝、航空物流等保税加工、保税物流、保税服务业，加快推进展览中心、如意全智能数字化纺纱等项目建设，打造加工贸易产业聚集区、国际物流分拨中转区、现代服务业创新区。兴庆区，整合通航产业园、商贸物流中心，重点发展商贸流通、通航运营、高端批发零售及服务业，打造消费经济牵引带、商贸物流集散地和国家通航产业示范区。金凤区，依托阅海湾中央商务区，重点发展总部经济、金融贸易、现代服务等核心产业，打造内陆开放型经济试验区核心区。西夏区，整合公铁物流园，深化与京东、普洛斯物流合作，完善现有基础设施网络和供应链，打造“一带一路”沿线综合性物流节点。永宁县，整合闽宁扶贫产业园、望远工业园，重点发展新能源轨道交通、生物发酵及制药产业，打造宁夏生物医药产业基地和闽宁对口协作产业聚集区。贺兰县，整合生态纺织园、德胜工业园、生物科技园，重点发展现代纺织、现代服务、特色农产品加工产业，打造现代纺织新城、特色农产品加工区。灵武市，整合高新技术开发区、再生资源循环经济示范区，重点发展现代纺织、再生资源循环利用及再制造产业，打造中国“羊绒之都”、国家“城市矿产”示范区。创新园区体制机制。在丝路经济园、中关村双创园率先引进专业化园区管理公司，启动市场化运营管理试点。分类制定差异化、个性化指标体系、政策体系、统计体系、考核评价体系，开展绩效考核、人事调配等配套改革试点，提升园区发展效益，打造经济发展主战场主引擎。

实施项目攻坚工程。坚持经济工作项目化，切实把握投速、投量、投向、投效，以投资驱动、项目拉动稳增长、调结构、添动力、促转型。深入研究项目。对标中央、自治区重大发展战略和重大项目包、资金包、政策包，围绕“两园三区”基础配套、“十大产业”延链补链、新兴产业集聚壮大和生态环保、民生改善等重点领域，认真研究、科学谋划、包装储备一批投资强度大、经济效益好、牵引作用强的重大项目，编制重大项目三年滚动投资计划，形成有特色、有针对性的梯次项目库，增强可持续发展后劲。加快项目建设。严格落实市级领导包抓和“五个一”项目推进机制，计划实施首批项目1030个，总投资3880亿元。重点抓好自治区在银实施的20个60大庆项目，推动天山·海世界一期、共享装备制造双创示范基地、瑞信葡萄生态旅游等项目建成投用。突出抓好市级15个60大庆项目，建成妇幼保健院产科大楼、紧急救援中心等项目。全力抓好“两园三区”基础设施等100个市级重点项目，力促一批重大项目建成投产。大力引进项目。坚持引资引智引技并举，开展战略招商、产业链招商、项目招商，实施央企和民企500强、军工企业、上市公司对接入银行动，引进一批10亿元以上大项目好项目，推动重大节会签约项目早落地、早建设、早见效。健全基金引导、高比例配资等招商引资机制，推行统计入库考核制度，将项目资金争取情况与部门预算、绩效考核挂钩，确保政策性项目资金增长10%以上。开展“投资环境提升年”活动。以自治区60周年大庆氛围营造、环境整治为契机，聚焦发展环境、市场发育体系的突出问题，统筹抓好优惠政策落实、政务环境优化等工作，切实完善相关政策，着力降低企业制度性交易成本和融资、电力、物流等生产经营成本，努力营造国际化便利化法治化投资环境。

实施科技强市工程。加快宁夏沿黄科技创新改革试验区、银川人才管理改革试验区建设，打造西部创新发展高地。搭建科技创新平台。依托银川产业技术研究院，深化与科技强省、知名院校、科研院所合作共建，实施一批重大科技创新项目，加快组建中国葡萄酒、上海交大新材料、环境生态、军民融合技术创新中心等产业研究院，建设5-7家科技成果转移转化研究机构，在北京中关村、苏州工业园等科技资源聚集地搭建3-5家离岸孵化器，推动科技成果就近就地孵化、项目银川转化。大力引进培养创新人才。坚持不求所有、但求所用，围绕主导产业编制尖端急需人才目录，依托创新创意在银川、大学生引进万人计划等品牌活动，柔性引进科技创新、现代金融、现代农业等方面高精尖缺人才600名左右、高层次创新团队15个以上；实施“五项人才工程”，培育一批科技领军人才、“银川工匠”和农村、社区适用人才。构建“亲”“清”新型政商关系，主动为非公经济和中小微企业搭好平台、搞好服务，全力打造一支具有战略眼光、全局视野、创新能力、善于经营的现代企业家队伍。营造风生水起的创新生态。探索知识产权综合管理改革，加强知识产权保护，完善科技创新成果运行激励、业绩分配、转化运用等机制，搭建科研成果交易服务平台。建立健全人才多元评价和激励体系，设立人才发展专项资金，完善人才认定、住房保障、支持创新创业等一揽子政策，启动高端人才社区建设，加大高端人才科研启动经费补助、研究成果奖励，让各类人才的创造力竞相迸发、聪明才智充分涌流。

实施改革开放驱动工程。深化重点领域关键环节改革，扩大开放范围和层次。深化“放管服”改革。加快全国行政审批标准化试点建设，全面推行“不见面、马上办”，力争80%进厅事项网上办理。深化“证照分离”，推进“多证合一、一照一码”等商事制度改革，让群众和企业少跑腿、好办事。积极稳妥推进综合行政执法改革，深化智慧化、网格化和跨部门、跨领域“双随机一公开”监管，努力做到放得更活、管得更好、服务更优。深化金融和财税改革。加大力度引进银行、证券、保险、基金等金融业态和各大金融机构、法人机构总部，争取国家进出口银行在银设立分行；加快组建地方法人银行、寿险公司、消费金融公司。设立中关村双创园、石墨烯等产业基金，争取培育1-2家A股上市企业、8家新三板挂牌公司。落实“营改增”等减税降费政策和水资源税、环保税改革，推进“先税后证”税收征管，涵养财源、增强财力。实行全口径预算管理和政府举债终身问责、倒查责任等制度，开展跨部门联合监管，严格规范政府举债行为，坚决遏制债务增量，稳步化解存量债务。加强金融机构内控制度建设。探索建立金融主体双向信用评级体系和“黑名单”制度，防范特色支柱产业龙头企业出现债务危机，严厉打击金融违法行为,守住不发生系统性区域性金融风险的底线。深化国资国企改革。有效整合国有资产，健全国企法人治理结构，推进混合所有制改革及员工持股试点。改革国企监管体制和财务管理机制，立制度、定底线、抓关键、强问责，完善国资监管机构投资风险、收益评估、责任追究等制度，实行重大投融资行为科学论证、依规评估和集中研究决策，提高国资保值增值能力。深入推进社保统筹、文化旅游、教育卫生等领域改革。拓展开放空间。深度融入“一带一路”倡议，加快内陆开放型经济试验区核心区建设，推进投资贸易便利化。夯实开放载体。完善综合保税区、滨河新区等重点园区、重要区域基础配套和产业协作功能，打造内引外联的强大引擎、招商引资的集聚平台。拓宽开放通道。陆上，推动银川公铁物流园海关监管区建设，申建铁路一类口岸，加快进口肉类（水果、种苗）、整车进口指定口岸建设，深化与连云港、威海等港口合作，提升银川至德黑兰双向货运班列开行质量。主动承接高铁时代多元化运输需求，加快银川火车站、空港等综合枢纽建设，规划建设轨道交通。畅通公铁物流中心、国际航空港、综合保税区运输通道，积极发展多式联运。空中，提升已开通的国际航班运载能力，新开至卡塔尔多哈、韩国仁川直达航线，优化加密国内航线网络布局，争取机场72小时过境免签等政策落地，积极推进离境退税试点，力争进出境快件突破20万件，打造区域航空枢纽。网上，申建国家跨境电子商务综合试验区，探索“平台服务+通关机制+境外配送”跨境电子商务模式，加快海外仓和进口产品分拨中心建设，拓展多层次网上国际贸易。深化对外贸易。复制自贸区创新做法，积极申请一般纳税人试点政策，优化企业通关环境。积极参与国际产能合作，加快中沙产业园建设，支持本地企业在境外建立资源、产业、研发基地和营销网络，加快优势特色产品、技术、服务对外出口。全年进出口总额增长10%以上。深化与京津冀、长三角、珠三角和宁蒙陕甘毗邻地区经济合作，吸引国内外资本、产业、人才、技术等发展要素向银川汇聚。

各位代表，高端是竞争力的体现。高端的事业源于创新的思维，呼唤尖端的人才，需要宽广的平台，推动高质量的发展。我们要以世界眼光审视高端，以博大胸怀谋划高端，以精尖科技创造高端，以集群产业孕育高端，以国际标准衡量高端，让高端成为银川大地的最强音、引领发展的加速器、造福百姓的新动能。

（三）聚焦和谐发展，开展“六项计划”，加快建设更具幸福感的城市。针对民生保障薄弱环节，要始终把人民对美好生活的向往作为我们工作的出发点和落脚点，坚持抓发展与惠民生相结合，深入实施脱贫富民战略，在发展中提高保障和改善民生水平。

开展精准脱贫计划。小康路上一个都不能少。坚持扶贫同扶智扶志相结合，发挥各级干部特别是驻村和帮扶干部的作用，创新群众工作方法，加强教育培训引导，着力激发贫困群众勤劳致富的内生动力。严格按照“六个精准”要求，突出“一村一策、一户一策、一人一策”，实施三年滚动脱贫行动计划，统筹推进“五个一批”，开展产业扶贫、金融扶贫、精神脱贫等“八个专项行动”，深化闽宁扶贫协作，重点实施10个扶贫产业，每村扶持壮大1-2个农业支柱产业，培育10家扶贫产业合作社、100名致富带头人，进一步提高扶贫小额贷款覆盖率。做好建档立卡贫困户社保兜底工作，全面完成“十三五”移民搬迁安置任务，确保8535名建档立卡贫困群众脱贫销号、16个贫困村脱贫，移民收入增长10%。完善脱贫退出、激励约束等机制，健全督查、通报、协调、问责等制度，扎实开展扶贫领域腐败和作风问题专项整治，严防“数字脱贫”“被脱贫”，确保脱真贫、真脱贫。

开展就业增收计划。就业是最大的民生，收入是群众最关心的大事。完善就业创业政策服务体系，突出抓好高校毕业生、农民工、困难人员等重点人群就业创业，搭建“互联网+”、创新工场、双创示范基地等孵化创新平台10个以上，以创业带动就业。开展职业技能培训1万人以上，转移农村劳动力就业8万人。购买公益性岗位1000个，新增城镇就业4.5万人,城镇登记失业率控制在4.0%以内。实施差异化收入分配激励政策，完善工资正常增长机制，进一步提高转移性、工资性、经营性和财产性收入，千方百计让老百姓的钱袋子鼓起来。

开展社会保障提升计划。社会保障是群众生存发展的最后防线。坚持兜底线、织密网、建机制，让人民群众老有所养、困有所助、危有所救。深化社会保险“五险合一”经办体制改革，在全区率先完成国家第二批社会保险经办标准化试点工作。深入推进按病种分值付费方式改革，控制医疗费用不合理增长。深化机关事业单位养老保险制度改革，推进职工医保异地结算和大病保险“一站式”服务，稳步提高城乡居民、城镇职工养老保险和医疗保险保障水平。实施“同舟计划”，将建筑企业农民工全部纳入工伤保险。大力发展残疾人和慈善公益事业。健全农村留守儿童、老年人和妇女关爱服务体系，做好城乡低收入群体救助帮扶工作。加快建立多主体供应、多渠道保障、租购并举的住房制度，积极发展住房租赁市场，加大棚改货币化安置力度，筹集棚户区改造房源8397套，改造城中村137.8万平方米、城市危旧房17.56万平方米，力争用两年时间完成城中村拆迁改造安置，让住房困难群众住上“暖心房”。

开展教育质量提升计划。切实把群众家门口学校办好、办出质量。深化“首都带首府”教育合作，推进优质教育资源提标扩面。新建续建二十九中、北师大银川学校等20所中小学、幼儿园，扩大普惠性民办幼儿园购买服务，着力解决“择校热”“大班额”问题。推广学校联盟、集团化办学、智慧教育，推动优质教育资源向农村延伸、义务教育由基本均衡向优质均衡转化，促进城乡教育一体化发展。普及高中阶段教育，扩大农村户籍学生免学费覆盖面。加强校企合作，推动现代职业教育产教融合，一次性就业率保持在95%以上。探索开展婴幼儿照护、儿童早期教育服务。推动民办教育、特殊教育健康发展。

开展健康银川建设计划。没有全民健康就没有全面小康。深化“首都带首府”医疗合作，加强人才培养、技术交流、医疗协作，提升银川现代医疗卫生服务水平。实施爱心护理院、第一人民医院滨河医院等项目，统筹智慧医疗、医联体、急救一体化建设。推进“三医”联动改革，落实药品“两票制”改革，推广预约诊疗、远程医疗、家庭医生等便民惠民措施，让群众在家门口享受优质的医疗服务。加强重大疾病防控，实施传统中医药传承创新工程。争创全国食品安全示范城市，加强食品药品安全监管，建设“智慧食安”监管网，构建食品安全追溯体系，让群众吃得更放心。完善慢行步道等健身基础设施，举办银川国际马拉松等赛事，大力开展全民健身活动，打造具有区域特色的健身休闲示范市。

开展文明城市巩固提升计划。全力推进深化全国文明城市建设三年提升工程。践行社会主义核心价值观，广泛开展理想信念教育，培育社会公德、职业道德、家庭美德、个人品德。加强诚信体系建设，健全守信激励和失信惩戒机制，共享应用失信人员信息，建设“信用银川”。加强公共文化服务体系建设，办好中国银川互联网电影节、文化创意节等活动，提升文化软实力。持续深化文明村镇、文明单位、文明家庭、文明校园及“最美社区”“最美乡村”等创建，选树“最美家庭”“最美银川人”等道德楷模。大力弘扬时代风尚，扎实开展“四送六进·文化惠民”“书香银川·银川书香”等活动，引领提升市民素质，让文明成为银川最靓底色。

各位代表，和谐是发展的基础。自然和则美，生命和则康，社会和则安，民族和则兴、国家和则强。唯有“和”才能 “合”，才能凝聚起同舟共济、合力攻坚的磅礴力量。全市上下要齐心协力，共同建设安全稳定、文明有序的和谐城市，让人民群众在和谐发展中不断增强获得感、幸福感、安全感。

（四）聚焦宜居发展，推进“四项行动”，加快建设西北地区重要中心城市。倍加珍视资源环境，着力推动城市精明增长，以银川都市圈为主体形态，推进大中小城市和小城镇、乡村协调发展，不断放大银川平原宜居宜业的独特优势，全面提升银川的宜居性和美誉度。

推进银川都市圈引领发展行动。制定银川都市圈建设实施意见，充分发挥龙头带动作用，加快建设创新绿色智慧宜居都市圈。深化产业分工协作。围绕都市圈五大优势产业集群，主动做好与石嘴山市、吴忠市、宁东在能源化工、纺织产业等类同领域的协作，逐步清出不符合银川发展定位的产业。加快重大基础设施互联互通。配合自治区抓好银西、包银高铁、乌银高速公路、国际航空港综合枢纽等重大项目建设，打通石嘴山-银川-青铜峡、艾依河至沙湖旅游快速通道，推进都市圈公交一卡通，构建“半小时经济圈”。统筹能源、供水、供暖、光纤等地下管网联通共享，推进区域天然气长输管线建设，实施都市圈城乡西线供水等项目，解决用气紧张和饮用水源不足问题。加强生态环境共保共治。制定实施银川都市圈三年城乡生态绿化行动计划，强化外围防沙治沙和荒漠化治理，加快建设“三大生态经济廊道”。建立环境保护联防联控机制，开展跨区域联合执法，保护好“母亲河”、守护好“父亲山”。推动公共服务共建共享。申办世界城市日全球主场活动，持续放大“教育在银川、医疗在银川”等品牌效应，服务和辐射带动区域发展。

推进城市功能提升行动。坚持精致规划、精心建设、精细管理，抓好“城市双修”、城市设计、新型城镇化等国家级综合试点，启动《银川城市总体规划（2018-2035）》编制工作，推进“兴庆率先”“金凤跨越”“西夏突破”，贺兰、永宁“两翼齐飞”，灵武与综保区、滨河新区、宁东融合发展，构建结构合理、分工明确、功能互补、产城融合的空间格局。健全完善工业园区、城镇、乡村低效用地评估机制，最大限度挖掘盘活城镇低效用地。创新长期租赁、租让结合、先租后让等多元供地方式，推动城市更新与产业深度融合、互促共进。实施凤凰北街打通、贺兰山西路拓宽改造等项目，改造提升10条小街巷，打通断头路，畅通微循环。新建一批停车场，切实解决交通拥堵问题。抓好垃圾分类示范城市建设，规划建设城市餐厨垃圾处理厂，改扩建一批小型垃圾转运站，配套建设20座公厕。壮大“银川眼”市民监督队伍，把精细化、精准化的管理延伸到城市的每个角落。

推进农业农村优先发展行动。制定乡村振兴三年行动计划，加快推进农业农村现代化，着力建设“产业兴旺、生态宜居、乡风文明、治理有效、生活富裕”的新农村。以产业振兴带动乡村振兴，加快构建现代农业“三大体系”，调整优化现代农业区域产业功能布局，延长产业链、提升价值链、拓展增收链，推动农业提质增效。建设三区都市型生态高效农业产业发展区、两县一市优势特色产业发展区，做精优质粮食产业，粮食生产功能区稳定在93万亩，做强草畜产业集群，做大做响优质蔬菜、花卉园艺、适水产业和都市创意休闲产业，大力发展休闲观光、田园综合体等乡村旅游，加快推进一二三产融合发展，千方百计增加农民收入。深化农业农村改革，落实“三权分置”政策，加快建立利益联结分享机制。突出“塞上江南”主题，打造一批特色小城镇、美丽乡村和美丽庭院。以“两处理、两改造”为抓手，持续推进农村基础设施配套和环境综合整治，推动城市供热、给排水、天然气、污水管网向农村延伸，开展农村“厕所革命”，力争卫生厕所普及率100%。提升农村绿化、环保、卫生等基础设施和公共服务配套标准，营造良好人居环境。

推进社会治理提升行动。优化智慧城市运行管理机制，加快推进互联网、物联网、大数据在政务服务、民生、城市治理等领域的应用，推动社会治理高效精准、惠民便民。加强“平安银川”建设，深化“雪亮工程”，严厉打击违法犯罪行为。坚持安全发展，健全公共安全体系，完善安全生产责任制，强化重点行业、领域排查治理，坚决遏制重特大事故发生。推进应急体系建设，提高突发事件应急处置和防灾减灾救灾能力。加强信访维稳工作，强化重大决策社会稳定风险评估，完善矛盾纠纷多元调解体系，最大限度减少不和谐因素。深入开展民族团结创建活动，争创全国民族团结进步示范市，依法加强宗教事务管理。深化军民融合，争创全国双拥模范城市九连冠。

各位代表，宜居是城市的永恒追求。城市让生活更美好，宜居让城市更集聚。建设宜居城市、美好家园，是每个人的向往。要把银川当作我们共同的家，精心地去规划它、建设它、经营它、呵护它，不断提升城市内涵、品质、实力，使人民群众生活得更方便、更暖心、更美好。

三、加强政府自身建设

打铁必须自身硬。我们要主动适应发展新形势、人民新期待，加快政府职能转变，不断提高科学施政水平，努力建设人民满意的政府。

建设学习型政府。始终把思想政治建设摆在首位，系统学习党的十九大精神和习近平新时代中国特色社会主义思想，扎实推进“两学一做”学习教育常态化制度化，积极开展“不忘初心、牢记使命”和“红船精神”等主题教育，增强“四个意识”，坚定“四个自信”，始终与以习近平同志为核心的党中央保持高度一致，确保思想不偏向、工作不走样。加强市政府党组工作，自觉贯彻执行民主集中制等各项制度，把党领导一切贯穿于政府工作全过程。着眼银川市长远发展，立足工作实际和岗位职责，深入开展学习型机关创建、“大走访、大调研”等活动，深化移动端、互联网等学习平台应用，及时掌握现代金融、市场经济、智能智慧、互联网科技等相关知识，推动知识结构、能力结构变革更新，增强政府系统科学发展、改革创新、群众工作、狠抓落实、驾驭风险的能力和水平。

建设法治型政府。始终把依法行政贯穿于工作全过程。完善权力清单、责任清单，加强动态监管，严格依照法定权限和程序行使权力、履行职责。进一步完善政府议事规则，严格落实重大事项报告制度和重大决策风险评估制度，推进重大行政决策程序立法和合法性审查，出台重大决策事项目录，落实行政决策后评估、责任追究及倒查机制，提升政府决策科学化、民主化、法治化水平。主动接受人大法律监督、政协民主监督，高质量办理人大议案建议和政协提案。全面推进政务公开，有效畅通舆论监督和社会监督渠道。进一步加强政府立法，修订环境保护、城乡建设等领域政府规章，强化规范性文件监督管理。深入开展“七五”普法，创建法治政府示范市。

建设效能型政府。执行力是政府行政的生命力。我们要始终和全市人民想在一起、干在一起，大力倡导“深、严、细、实”作风，积极弘扬“钉钉子”“马上办”精神，建立“年初建账、年中查账、年底交账”工作推进机制，实行重点任务清单管理、销号认定、全程监督，强化政务督查、履职问责，全面提升行政效能。对标“四风”问题十种新表现，扎实开展“学查改”作风整治活动，以更高标准、更严举措正风肃纪，严防“四风”反弹回潮。进一步健全绩效考核体系，完善正向激励和容错纠错机制，旗帜鲜明为改革创新者撑腰鼓劲，激励广大公职人员担当实干，做出让人民群众满意的实绩。

建设廉洁型政府。始终把全面从严治党的各项要求落实到政府工作的各个环节，认真履行党风廉政建设责任制和“一岗双责”，严格执行《准则》《条例》，深入落实中央八项规定精神、国务院“约法三章”和区市若干规定，凡是群众反映强烈的问题都要严肃认真对待，凡是损害群众利益的行为都要坚决纠正。完善权力运行制约监督机制，深化领导干部经济责任审计，推进国资国企、脱贫攻坚、工程招投标等重点领域廉政监督和审计监督全覆盖，让权力在阳光下运行。牢固树立过紧日子的思想, 全面推行预算绩效管理评价，严控一般性支出,严禁铺张浪费。倡导“100-1=0”的廉洁从政理念，教育公职人员知敬畏、存戒惧、守底线，无禁区、全覆盖、零容忍查处腐败问题，切实做到干部清正、政府清廉。

各位代表，使命重在担当，实干赢得未来。政府是党的路线的坚定执行者、落实者，是人民利益的坚定维护者、实现者。站在新起点、展望新发展，让我们更加紧密地团结在以习近平同志为核心的党中央周围，在自治区党委、政府和市委的坚强领导下，始终把人民群众对美好生活的向往装在心里、抓在手上、落实到行动中，不忘初心、牢记使命，攻坚克难、砥砺奋进，为在全区率先全面建成小康社会、率先开启全面建设社会主义现代化新征程而努力奋斗！
